# Supplementary material for: Psychological Determinants of COVID-19 Vaccine Acceptance among Healthcare Workers in Kuwait: A Cross-Sectional Study Using the 5C and Vaccine Conspiracy Beliefs Scales
Source: Vaccines (Basel). 2021 Jun 25;9(7):701. doi: 10.3390/vaccines9070701 (PMC8310287; doi:10.3390/vaccines9070701)
Supplement: Supplementary file 1 [file vaccines-09-00701-s001.zip › vaccines-1265766-supplementary.pdf]

Supplementary S1

Consent form and questionnaire

مستوى قبول لقاحات كوفيد ١٩ بين العاملين في مجال الرعاية الصحية في الكويت

Covid-19 vaccine acceptance among healthcare workers in Kuwait

تم إعداد هذا الاستبيان لقياس موقف العاملين في مجال الرعاية الصحية في الكويت تجاه لقاح فيروس كورونا (كوفيد 2019). سيتم استخدام المعلومات المقدمة لك من خلال هذا الاستبيان لأغراض البحث فقط ، وسيتم التعامل مع البيانات بسرية تامة مع حماية كاملة لخصوصيتك.

المشاركة في هذا الاستطلاع تطوعية تمامًا.

متوسط الوقت اللازم لإكمال الاستبيان هو 5 دقائق فقط.

شكرًا جزيلاً على موافقتك على المشاركة في هذا الاستبيان.

This questionnaire has been prepared to measure the attitude of healthcare workers in Kuwait towards coronavirus disease 2019 (COVID-19) vaccine. The information provided to you through this questionnaire will be used for research purposes only, and the data will be treated with complete confidentiality with complete protection of your privacy.

Participation in this survey is completely voluntary.

The average time to complete the questionnaire is only 5 minutes.

Thank you very much for agreeing to take part in this survey.

If you agree to participate, please tick the next box **إذا كنت توافق على المشاركة ، يرجى اختيار المربع التالي**

I agree **أنا موافق**

---

1. Age العمر

………………….

2. Sex الجنس

Male ذكر

Female أنثى

3. Nationality الجنسية

………………….

4. Occupation المهنة

Physician طبيب/طبيبة

Dentist طبيب أسنان/طبيبة أسنان

Pharmacist صيدلاني/صيدلانية

Nurse ممرض/ممرضة

Laboratory technician فني/فنية مختبر

Other وظيفة أخرى

5. Educational level المستوى التعليمي

Undergraduate degree (diploma, BSc degree) دبلوم ، درجة البكالوريوس

Postgraduate degree (MSc, PhD degrees) ماجستير أو دكتوراه

6. Workplace مكان العمل

Public sector قطاع عام

Private sector قطاع خاص

7. Do you suffer from any chronic diseases (such as allergy, diabetes, hypertension or heart disease)? هل تعاني من أي أمراض مزمنة (مثل الحساسية أو السكري أو ارتفاع ضغط الدم أو أمراض القلب)؟

Yes نعم

No لا

8. Have you or any of your family members had a diagnosis of COVID-19? هل تم تشخيصك أنت أو أي من أفراد عائلتك بكوفيد ١٩؟

Yes نعم

No لا

---

9. Did you get COVID-19 vaccine/Do you intend to get COVID-19 vaccine? هل حصلت على لقاح كوفيد ١٩ / أو هل تنوي الحصول على لقاح كوفيد ١٩؟

Yes نعم

No لا

Maybe ربما

10. Do you oppose vaccination altogether? هل تعارض التطعيم واللقاحات تماماً؟

Yes نعم

No لا

No opinion ليس عندي رأي

11. What is your belief about the origin of the current coronavirus in humans? ما هو اعتقادك بشأن مصدر فيروس كورونا المستجد الحالي في البشر؟

Natural source from animals مصدر طبيعي من الحيوانات

Man-made virus and part of a conspiracy plot فيروس من صنع الإنسان وجزء من مؤامرة

No opinion ليس عندي رأي

---

12. Based on technology, which one of the following COVID-19 vaccines would you most likely accept? بالاعتماد على تكنولوجيا صناعة اللقاح، أي من لقاحات كوفيد ١٩ التالية ستقبلها على الأرجح؟

mRNA (messenger RNA) vaccine تلقيح الحمض النووي الريبوزي المرسال

adenovirus viral vector vaccine لقاحات الناقل الفيروسي الغديّ

inactivated SARS-CoV-2 vaccine اللقاحات المقتولة

sub-unit (virus-like particle) لقاح الوحدة الفرعية

13. Based on vaccine developer, which one of the following COVID-19 vaccines would you most likely accept? بالاعتماد على الشركات المطورة للقاحات ، أي من لقاحات كوفيد ١٩ التالية ستقبلها على الأرجح؟

Pfizer-BioNTech COVID-19 vaccine (US) (فايزر بيونتيك (أمريكي

Sinopharm COVID-19 vaccine (China) (لقاح سينوفارم (الصين

Oxford-AstraZeneca COVID-19 vaccine (UK/Sweden) لقاح أكسفورد–آسترازينيكا (بريطاني- سويدي)

Sputnik V COVID-19 vaccine (Russia) (لقاح سبوتنيك (روسيا

Moderna COVID-19 vaccine (US) (لقاح موديرنا (أمريكا

Johnson & Johnson COVID-19 vaccine (US/Belgium/Netherlands) لقاح جونسون آند جونسون (أمريكا-بلجيكا/هولندا)

14. What is the main source of information about COVID-19 vaccination? ما هو المصدر الرئيسي للمعلومات حول التطعيم ضد مرض كوفيد -١٩؟

TV programs, newspapers and news releases البرامج التلفزيونية والصحف والنشرات الإخبارية

Social media platforms (Facebook, Twitter, etc.)/YouTube منصات التواصل الاجتماعي (فيسبوك ، تويتر ، إلخ) / يوتيوب

Scientists/scientific journals العلماء / المجلات العلمية

Doctors/other healthcare workers الأطباء / غيرهم من العاملين في مجال الرعاية الصحية

**Item**	*Strongly Disagree أرفض بشدة*	*Disagree أرفض*	*Somewhat Disagree أرفض إلى حد ما*	*Neutral محايد*	*Somewhat Agree أوافق إلى حد ما*	*Agree أوافق*	*Strongly Agree أوافق بشدة*
15. Regarding vaccines, I am confident that public authorities decide in the best interest of the community فيما يتعلق باللقاحات ، أنا واثق من أن السلطات العامة تقرر المصلحة الفضلى للمجتمع							
16. Vaccinations are effective اللقاحات تعتبر فعالة							
17. COVID-19 is not so severe that I should be vaccinated مرض كوفيد ١٩ ليس شديدًا للدرجة التي تدفعني لأخذ اللقاح							
18. My immune system is so strong; it also protects me against COVID-19 جهازي المناعي قوي جداً. كما أنه يحميني من مرض كوفيد ١٩							
19. For me, it is inconvenient to be vaccinated بالنسبة لي ، فإن أخذ اللقاح هو أمر غير مريح							
20. Visiting the doctor makes me feel uncomfortable; this keeps me from being vaccinated زيارة الطبيب تجعلني أشعر بعدم الارتياح. هذا يمنعني من التطعيم							
21. For each and every vaccination, I closely consider whether it is useful for me بالنسبة لكل تطعيم ، أفكر جيدًا فيما إذا كان مفيدًا لي أم لا							
22. It is important for me to fully understand the topic of vaccination before I get vaccinated من المهم بالنسبة لي أن أفهم تمامًا موضوع التطعيم قبل أن أتلقى التطعيم							
23. I get vaccinated because I can also protect people with a weaker immune system أتلقى التطعيم لأنني بذلك أستطيع أيضًا حماية الأشخاص الذين يعانون من ضعف في جهاز المناعة							

24. Vaccination is a collective action to prevent the spread of diseases التطعيم هو عمل جماعي لمنع انتشار الأمراض							

Item	*Strongly Disagree أرفض بشدة*	*Disagree أرفض*	*Somewhat Disagree أرفض إلى حد ما*	*Neutral محايد*	*Somewhat Agree أوافق إلى حد ما*	*Agree أوافق*	*Strongly Agree أوافق بشدة*
25. COVID-19 vaccine safety data is often fabricated بيانات سلامة لقاح كوفيد غالبًا مُزيفة							
26. Immunizing children is harmful, and this fact is covered up تطعيم الأطفال ضار وهذه الحقيقة محجوبة عن الناس							
27. Pharmaceutical companies cover up the dangers of COVID-19 vaccines تُخفي شركات الأدوية عن الناس مخاطر لقاح كوفيد ١٩							
28. People are deceived about COVID-19 vaccine efficacy يتم خداع الناس بشأن فعالية لقاح كوفيد ١٩							
29. Vaccine efficacy data is often fabricated غالبًا ما تكون بيانات فعالية اللقاحات مُزيفة							
30. People are deceived about vaccine safety يتم خداع الناس بشأن سلامة اللقاحات							
31. The government is trying to cover up the link between vaccines and autism تحاول الحكومة التستر على الصلة بين اللقاحات وأمراض أُخرى مثل التوحد							

Thank you very much for participating in the survey شكرا جزيلا لك على المشاركة في الاستبيان
